# Differential Reporting of Adolescent Stress as a Function of Maternal Depression History

**DOI:** 10.1007/s10608-014-9654-4

**Published:** 2014-11-05

**Authors:** Issar Daryanani, Jessica L. Hamilton, Benjamin G. Shapero, Taylor A. Burke, Lyn Y. Abramson, Lauren B. Alloy

**Affiliations:** 1 Department of Psychology, Temple University, 1701 N. 13th St., Philadelphia, PA 19122 USA; 2 Department of Psychology, University of Wisconsin, Madison, 1202 W. Johnson St., Madison, WI 53706 USA

**Keywords:** Maternal depression, Report discrepancy, Adolescence, Stress

## Abstract

The depression–distortion hypothesis posits that depressed mothers report child characteristics in a negatively-biased manner, motivating research on discrepant reporting between depressed mothers and their children. However, the literature has predominately focused on report discrepancies of youth psychopathological and behavioral outcomes, with limited focus on youth stress despite the marked increase of stressful events during adolescence. The current study investigated whether the presence versus absence of a maternal history of major depressive disorder differentially influenced reporting of adolescent stress when compared to her child’s report, utilizing a community sample of diverse adolescents. As hypothesized, mothers with a history of depression were more likely to report more youth stress than their children reported. Specifically, mothers with a history of depression were more likely than nondepressed mothers to report more familial, social, and youth-dependent stressors relative to their children; nondepressed mothers were more likely to report less independent stressors than their children.

## Introduction

Depression is associated with pervasive impairment in multiple facets of life, and can be debilitating due to its severity (McDermott and Ebmeier 2009), recurrent nature (Vittengl et al. 2007), and high rate of comorbidity (Mineka et al. 1998). The high prevalence of depression further exacerbates its destructive effect, at both personal and societal levels. In adults, the prevalence of depression in women is roughly twice as high as in men (Kessler 2006). Additionally, there is a notable increase in incidence of depression during adolescence, a period marked by stressful events, concerns about self-image, and peer victimization (Ge et al. 1994; Hankin et al. 1998; Prinstein et al. 2001). Thus, women and adolescents are at particular risk for developing depression.

Women and adolescents’ elevated rates of depression create the potential for a vicious storm, as it is well documented that maternal depression can have many negative effects on youth, particularly during the vulnerable period of adolescence (Beardslee et al. 1998; Downey and Coyne 1990; Goodman et al. 2011). Maternal depression has been shown to be predictive of youth depression (Goodman and Gotlib 1999; Hammen et al. 2004), higher levels of stressful events among youth (Compas et al. 2002), and youth social adjustment problems (Breslau et al. 1988). Further, negative cognitive styles typically consolidate during adolescence, and exposure to mothers’ negative cognitive styles during adolescence may lead to the development of similar maladaptive styles in offspring (Goodman and Gotlib 1999; Hankin 2008).

Consequently, understanding the relationship between depressed mothers and their adolescents is important; unfortunately, reliably studying this relationship has been problematic because of inherent discrepancies when multiple informants assess the same construct (for a review, see De Los Reyes and Kazdin 2005). Although variance in discrepant reporting can be partially attributed to random error (De Los Reyes 2011) and the reporter’s environment (Achenbach et al. 1987), a substantial portion of the variance may be accounted for by reporter characteristics (Briggs-Gowan et al. 1996). As researchers often attempt to consolidate information from mothers and their children, maternal characteristics such as depression have been investigatory priorities in the discrepancy literature.

A majority of research that examines the influence of maternal depression on discrepant reports with their children is informed by the depression–distortion hypothesis. The depression–distortion hypothesis (Richters and Pellegrini 1989) posits that maternal depression is associated with negatively-biased discrepancies when reporting on child characteristics, a discrepancy not observed in nondepressed mothers. Depressed mothers have exhibited negatively-biased reporting discrepancies of youth behavioral problems (Chi and Hinshaw 2002; Youngstrom et al. 1999), depressive symptoms (Richters 1992; Youngstrom et al. 2000), and social interactions (Youngstrom et al. 1999) relative to their children. Further, it may be misleading to assume that nondepressed individuals are accurate reporters. Although not studied explicitly in the context of mother–youth discrepancies, nondepressed individuals have exhibited optimistic cognitive biases, through which they overestimate their control over events, provide self-enhancing judgments about their successes and personal characteristics, and exhibit a bias to recall events more favorably than what objectively occurred (Alloy and Abramson 1979, 1988; Taylor and Brown 1988). Maternal depression’s role in reporting discrepancies between mothers and their children remains an understudied topic, as it has almost exclusively focused on youth psychopathological and behavioral outcomes.

Surprisingly, the depression–distortion hypothesis has yet to be investigated in the context of youth stress, which warrants attention given the increase in stressful events youth report experiencing during adolescence (Ge et al. 1994). A thorough examination entails investigating stress as more than a singular construct, as specificity of the type of stressor is considered an important distinction when studying negative adolescent life events. Depression research often distinguishes between independent stressors (events that an individual has no contribution to, such as natural disasters) and dependent stressors (events that are at least partially attributed to the behavior of an individual, such as peer conflicts; Hammen 2005). Stressful life events in general are a risk factor for depression (Monroe and Harkness 2005), but dependent life events are more strongly associated with depression onset and maintenance (Liu and Alloy 2010), and depressed individuals experience more episodic stress than nondepressed individuals due to personal characteristics (Hammen 2005). More generally, negative life events can also be investigated by content-domains (e.g., family, achievement, social, financial, legal, etc.), and different domains of life stress have differing influences on psychopathology (for a review, see Monroe and Simons 1991).

The increased risk of psychopathology and maladjustment for adolescents with a depressed mother is disheartening. Reporting discrepancies between mothers and their children exacerbate the problem by making it harder to ascertain adjustment problems children are facing; hence, it becomes difficult to reliably study the mother–youth relationship and subsequently discern mechanisms of intergenerational psychopathology transmission. No study to our knowledge has examined whether mothers with a history of depression differentially acknowledge their adolescents’ stressful experiences compared with nondepressed mothers. Given the importance of making specific distinctions within the stress literature, there is great utility in deriving a better understanding of how inter-reporter discrepancies manifest based not only on overall youth stress reporting, but also on the type of stressor.

## Hypotheses

We hypothesized that mothers with a history of major depressive disorder (MDD) would differ in their reporting of adolescent stress from nondepressed mothers. Specifically, mothers with a history of MDD would be more likely to report more stressors in their adolescent children’s lives than their children’s reports, and mothers with no history of depression would be more likely to report fewer stressors in their children’s lives than their children’s reports. The hypothesis was informed by the depression–distortion hypothesis (Richters and Pellegrini 1989), such that depressed mothers are posited to show negatively-biased report discrepancies with their children, which we believed would be congruent with an increased reporting of youth negative life events. If our hypotheses were supported and there were significant group differences, we proposed to further explore whether the tendency for mothers to over- or under-report relative to their children’s reports differed by the domain of the stressor (Academic, Family, and Social life events) and the dependence of the events (Independent of versus Dependent on the child).

## Methods

### Participants

The sample consisted of 300 mother–youth dyads who participated in the Adolescent Cognition and Emotion (ACE) Project, a prospective longitudinal study of adolescent development and risk for depression (see Alloy et al. 2012 for detailed sample description). The project recruited adolescents and their primary female caregivers (collectively referred to as “mothers” because 93 % were biological mothers) from Philadelphia-area middle schools. Inclusion criteria specified that adolescents be ages 12–13 at baseline, self-identify as white or black, and have a mother who lives with the child and can participate in the study. Excluded dyads included mothers or adolescents who did not speak or read English sufficiently to participate, or had any history of psychosis, severe cognitive impairment, or medical problems that would substantially hinder completion of the study measures.

Adolescents (N = 300, M = 12.83 years, SD = . 61 years) were evenly represented across gender (46 % male, 54 % female) and self-identified race (52 % African American, 48 % Caucasian). Approximately 47 % of the adolescents qualified for subsidized lunch at their middle schools, an indicator of low socioeconomic status that takes into account the number of dependents supported on the family’s income. The mothers in the sample varied in age (M = 42.07 years, SD = 7.07 years) and years of education (M = 14.50, SD = 2.53). Mothers were assessed at baseline for a history of MDD, with 31.7 % (N = 95) meeting criteria at some point in their past.

### Procedure

After a screening phone call to determine eligibility and project interest, participants were invited for a baseline visit. Written consents and assents were obtained from mothers and adolescents, respectively, at the beginning of the visit. During the baseline visit, mothers provided demographic information and were assessed for a history of MDD using a semi-structured diagnostic interview. At the follow-up visit, approximately 9 months after baseline, mothers and their children separately identified the occurrence of negative life events in the youths’ lives since baseline. Mothers and their adolescents also were assessed for current depressive symptoms and diagnoses at follow-up.

### Measures

#### Youth Stress

The Adolescent Life Events Questionnaire (ALEQ; Hankin and Abramson 2002) is a self-report scale that assesses the occurrence and severity of 63 negative life events common during adolescence. Adolescents and their mothers completed separate versions of the measure at the follow-up visit, indicating negative events that had happened in the adolescents’ lives since the baseline visit (both the parent report and youth report assess the same events). The items probe events that may have occurred in various domains, including family, school, peer, relationship, and body image stressors. We utilized three domains in analyses, in addition to a total stressor score: Academic (school), Family, and Social (peer, relationship, and body image stressors). Additionally, life events were categorized a priori as either Dependent (e.g., “You had a fight with your friend”; 42 total items) or Independent (e.g., “Your parent(s) had financial troubles”; 21 total items) for the child. Four clinical psychology doctoral students rated the events as either dependent or independent, achieving an inter-rater reliability of κ = .76. When discrepancies were found, a consensus was arrived at after further discussion. The ALEQ has been examined as a reliable and valid measure of adolescent stress (Hankin 2008; Hankin and Abramson 2002).

#### Maternal Depressive Diagnoses

The Schedule for Affective Disorders and Schizophrenia-Lifetime (SADS-L; Endicott and Spitzer 1978) is a semi-structured clinical interview used to diagnose adult psychopathology. For the ACE project, an expanded version of the SADS-L was employed to accurately determine criteria for Axis I disorders, as per the *Diagnostic and Statistical Manual of Mental Disorders* (4th ed., text rev.; *DSM*-*IV*-*TR*; American Psychiatric Association 2000). Modifications of the SADS-L for our project included additional depressive symptom probes to allow for assessment of *DSM*-*IV*-*TR* criteria, and a more thorough assessment of the duration and frequency of depression. Diagnosticians included postdoctoral fellows, clinical psychology doctoral students, and post-baccalaureate research assistants, all of whom completed a 200-h intensive training program. Inter-rater reliability, when compared with an expert psychiatric diagnostic consultant, yielded κ = .86 (based on 100 expanded SADS-L interviews). The mothers in the project were assessed with the expanded SADS-L at the baseline visit, and those in the depressed group met criteria for MDD at some point in their lives. At the follow-up visit, mothers were administered the SADS again to assess for any current diagnoses of MDD, used as a covariate in primary analyses.

#### Youth Depressive Diagnoses

The Kiddie-Schedule for Affective Disorders and Schizophrenia-Epidemiological Version (K-SADS-E; Orvaschel 1995) is a clinical interview that assesses psychopathology in children and adolescents, as per the *DSM*-*IV*-*TR*. Postdoctoral fellows, clinical psychology doctoral students, and post-baccalaureate research assistants conducted the interviews following an intensive training program. Inter-rater reliability based on 120 pairs of ratings (ten interviews with 24 total diagnoses, rated by five interviewers) was *κ* = .85. An adolescent met for a “Depressive Disorder” if they currently met criteria for major depressive disorder or sub-threshold major depressive disorder at follow-up. Sub-threshold diagnoses were given if an adolescent endorsed five or more depressive symptoms for greater than 1 week but less than 2 weeks, or endorsed three or four symptoms for at least 2 weeks.

#### Maternal Depressive Symptoms

The Beck Depression Inventory (BDI; Beck et al. 1978) is a self-report questionnaire used to assess depressive symptoms in adults over the past 2 weeks. The 21-item inventory, often employed for its strong psychometric properties (Beck et al. 1988), was administered at follow-up to mothers to control for their current depressive symptoms. Cronbach’s alpha in our study yielded α = .82.

#### Youth Depressive Symptoms

The Children’s Depression Inventory (CDI; Kovacs 1985) is a widely utilized, 27-item questionnaire administered to children and adolescents to assess depressive symptoms. The youth rates each item on a zero to two scale based on how he or she has been feeling during the past 2 weeks, with overall scores ranging from zero to 54 (higher scores indicate the presence of more depressive symptoms). The CDI has demonstrated an ability to validly measure depressive symptoms in youth samples (Klein et al. 2005). The CDI was administered at follow-up to control for youth depressive symptoms in all primary analyses. Cronbach’s alpha for our study yielded α = .85.

## Results

### Computation of Discrepancy Scores between Mothers and Adolescents

To examine whether there was a significant difference in reporting of adolescent stressful life events by mothers with and without a history of depression, as compared to their adolescents, we computed discrepancy scores. First, we summed the total number of events that were reported by mothers and not by adolescents (termed “over-reported” events by the mother). Second, we summed the total number of events that were reported by the adolescent and not by the mother (termed “under-reported” events by the mother). We then computed over- and under-report variables for independent/dependent and domain-specific (Family, Academic, and Social) negative life events. Items that the mother and child agreed upon as either occurring or not occurring were considered non-discrepant. All discrepancies were relative to the child report; we could not explicitly investigate the presence of maternal report biases, as no objective measure of the occurrence of stressful events was available.

We also calculated an absolute value of total discrepancy. To do this, we summed the total number of over-reported and under-reported events across all event types. Thus, higher scores reflect a greater number of unmatched events between mothers and their children (e.g., mother reported and adolescent did not or adolescent reported and mother did not). This calculation was performed to identify whether mothers with and without a history of depression differed in overall acknowledgment of their adolescents’ stressful life events.

### Descriptive Analyses

Descriptive statistics for demographic and primary study variables are presented by maternal diagnosis in Table 1. Analyses were conducted to determine whether there were any significant differences on demographics (gender, race, SES based on eligibility for free lunch, age) and primary outcome variables. Compared to mothers without a history of depression, mothers with a history of depression were significantly older (*t* = 2.09, *p* < .05), and reported more events on the ALEQ (*t* = 3.08, *p* < .01). Additionally, adolescents whose mothers had a history of maternal depression were significantly more likely to be Caucasian (χ^2^ = 10.85, *p* < .001), but were not more likely to report events on the ALEQ (*t* = −.71, *p* = .48). Mothers with a history of depression were no more likely to meet criteria for current MDD (χ^2^ = .98, *p* = .25) or experience depressive symptoms (*t* = 1.00, *p* = .32) than nondepressed mothers at follow-up.

**Table 1:** Descriptive statistics and differences in variables by maternal depression history

Characteristics	History of MDD	No History of MDD
*n*	95	205
Youth age	12.85 (.64)	12.83 (.60)
Youth gender (female)	51 (54 %)	105 (51 %)
Youth race (AA)	36 (38 %)***	119 (58 %)
Free lunch	50 (53 %)	105 (51 %)
Mother age	43.33 (7.28)*	41.49 (6.92)
CDI	7.43 (7.20)	6.68 (5.50)
Youth depressive Dxs	11 (12 %)	14 (7 %)
BDI	6.41 (5.61)	5.09 (5.53)
Maternal MDD follow-up	5 (5 %)	6 (3 %)
Maternal total ALEQ report	15.26 (7.91)**	12.41 (6.42)
Youth total ALEQ report	14.77 (8.12)	14.08 (7.70)
ALEQ absolute discrepancy	13.83 (5.67)	13.22 (5.19)
Total over-report	7.17 (5.08)**	5.76 (4.16)
Total under-report	6.60 (4.95)	7.41 (5.25)
Family over-report	3.83 (2.74)*	3.16 (2.51)
Family under-report	2.22 (1.93)	2.70 (2.37)
Academic over-report	1.36 (1.49)	1.12 (1.24)
Academic under-report	1.64 (1.44)	1.83 (1.52)
Social over-report	1.96 (2.02)*	1.38 (1.70)
Social under-report	2.57 (2.68)	2.73 (2.59)
Indep over-report	1.47 (1.54)	1.16 (1.19)
Indep under-report	1.36 (1.49)*	1.91 (1.98)
Dep over-report	5.72 (4.35)*	4.50 (3.59)
Dep under-report	5.08 (4.07)	5.41 (4.11)

Means are presented with standard deviations in parenthesis, where applicable

*MDD* major depressive disorder, *AA* African American, *CDI* Children’s Depression Inventory, *BDI* Beck Depression Inventory, *ALEQ* Adolescent Life Events Questionnaire, *Dxs* diagnoses, follow-up, *Indep* independent events, *Dep* dependent events

* *p* < .05; ** *p* < .01; *** *p* < .001

Table 2 displays bivariate correlations of primary study variables. As expected, maternal depressive diagnosis at follow-up (i.e., current MDD) was positively correlated with current BDI; similarly, youth follow-up depressive diagnoses were positively correlated with follow-up CDI scores. Further, ALEQ total over-report was negatively correlated with ALEQ total under-report, suggesting that mothers who tended to over-report items on the ALEQ relative to their children were less likely to under-report on other items (Table 2).

**Table 2:** Bivariate correlations between study variables

	1	2	3	4	5	6	7	8	9	10	11
1. Maternal MDD history	–										
2. CDI	.06	–									
3. Youth depressive Dxs	.08	.19**	–								
4. BDI	.11	.07	.09	–							
5. Maternal MDD follow-up	.06	.20***	.20***	.12*	–						
6. ALEQ total over-report	.15**	.10	.09	.05	.11	–					
7. ALEQ total under-report	−.07	.11	.02	−.08	.04	−.40***	–				
8. ALEQ Indep over-report	.11	.11	.05	.01	.03	.59***	−.19**	–			
9. ALEQ Indep under-report	−.14*	.11	−.01	−.06	.05	−.24***	.70***	−.18**	–		
10. ALEQ Dep over-report	.15*	.08	.10	.03	.12*	.96***	−.40***	.34***	−.21***	–	
11. ALEQ Dep under-report	−.04	.09	.02	−.05	.03	−.41***	.94***	−.16**	.42***	−.42***	–

*MDD* major depressive disorder, *CDI* Children’s Depression Inventory, *BDI* Beck Depression Inventory, *ALEQ* Adolescent Life Events Questionnaire, *Dxs* diagnoses, follow-up, *Indep* independent events, *Dep* dependent events

* *p* < .05; ** *p* < .01; *** *p* < .001

The number of events reported by adolescents and their mothers, as well as all discrepancy outcome variables, were normally distributed and homogenous in their variances across groups.

### Total Discrepancy between Adolescents and Mothers on the ALEQ

To examine whether the total discrepancy in reported life events between the adolescents and their mothers was different based on maternal depression history, we first conducted *t* tests. Results suggested that there were significant differences on total over-report discrepancy scores between mothers with and without a depression history, such that mothers with a history of depression were significantly more likely to over-report events on the ALEQ than mothers without a history of maternal depression (*t* = 2.54, *p* = .01). However, there was no significant difference in the extent to which mothers under-reported (*t* = 1.27, *p* = .21).

To compare the overall absolute value of discrepancy of mothers with and without a depression history, we conducted a *t* test on the absolute value of total over- and under-reported events. These results revealed that there were no overall differences in the extent to which mothers with and without a depression history reported the same events as their children (*t* = .91, *p* = .36). Thus, this indicated that mothers with and without a depression history were similarly discrepant in magnitude from their children, but in different directions.

Second, to determine whether maternal history of depression significantly predicted over-report discrepancy differences independent of our designated covariates (youth depressive symptoms and diagnoses at follow-up, maternal depressive symptoms and diagnoses at follow-up, youth gender, and total number of youth-reported events), we conducted hierarchical linear regressions (Table 3). In Step 1, all covariates were entered predicting to over-report discrepancy; in Step 2, history of maternal MDD was included in the model. Results indicated that, independent of covariates, mothers with a history of MDD exhibited greater over-reporting on the ALEQ than nondepressed mothers (β = .14, *t* = 2.50, *p* = .01, Δ*R* ^2^ = .02, *f* ^2^ = .02). Although the covariates alone accounted for 8.5 % of the variance in over-reporting of events, maternal history of MDD explained a significant 2 % of additional variance (Table 3). As total under-report discrepancy was not significantly different between groups, we did not conduct further analyses.

**Table 3:** Hierarchical regressions predicting over-report discrepancy between mothers and adolescents on stressful life events

Step	Variable	β	*t*	*Model R* ^*2*^	*Model F*	*f* ^2^
*Total events*						
Step 1	CDI follow-up	.14	1.88	.09	4.34***	.10
	Youth depressive Dxs	.05	.82			
	BDI follow-up	.03	.40			
	Maternal depressive Dxs	.09	1.52			
	Gender	−.01	−.05			
	Youth total report	−.27	−4.14***			
Step 2	Maternal MDD history	.14	2.50**	.11	4.68***	.02
*Dependent events*						
Step 1	CDI follow-up	.09	1.19	.08	4.16**	.09
	Youth depressive Dxs	.06	1.01			
	BDI follow-up	.06	.73			
	Maternal depressive Dxs	.11	1.82			
	Gender	.02	.32			
	Youth dependent report	−.27	−4.14***			
Step 2	Maternal MDD history	.14	2.45*	.10	4.49***	.02

MDD history and depressive Dxs are coded with 0 (absent) and 1 (present); gender is coded with 0 (male) and 1 (female)

*MDD* major depressive disorder, *CDI* Children’s Depression Inventory, *BDI* Beck Depression Inventory, *Dxs* diagnoses, follow-up

* *p* < .05; ** *p* < .01; *** *p* < .001

### Domain-Specific Event Discrepancy between Adolescents and Mothers on the ALEQ

Subsequently, we investigated the presence of domain-specific discrepancy differences between mothers with and without a history of MDD. Mothers with a history of MDD were more likely to over-report Family (*t* = 2.12, *p* = .04) and Social (*t* = 2.57, *p* = .01), but not Academic (*t* = 1.49, *p* = .14), stressors relative to nondepressed mothers. Reflecting the absence of significant differences on ALEQ total under-reporting between mothers with a history of MDD versus no history, there were no group differences on under-reporting of ALEQ Family items (*t* = 1.75, *p* = .08), Academic items (*t* = 1.03, *p* = .31), or Social items (*t* = .50, *p* = .62).

Hierarchical linear regressions were conducted to determine whether the tendency for mothers with a history of MDD to over-report Family and Social events remained significant while controlling for: maternal depressive symptoms and diagnoses, adolescent depressive symptoms and diagnoses, youth gender, and youth reported events. For domain-specific analyses, youth total reported events were divided into subcategories based on domain (e.g., if an adolescent endorsed 20 negative life events overall, but only 8 within the domain of “Social” stressors, the “youth report” covariate inputted for analyses of Social over-report would equal 8). Independent of covariates, a history of maternal MDD predicted a significantly greater degree of over-reporting of stressors in the Family (β = .12, *t* = 2.05, *p* = .04, *R* ^2^ = .13, Δ*R* ^2^ = .01, *f* ^2^ = .01) and Social (β = .13, *t* = 2.26, *p* = .03, *R* ^2^ = .09, Δ*R* ^2^ = .02, *f* ^2^ = .02) domains.

### Independent and Dependent Event Discrepancy between Adolescents and Mothers on the ALEQ

Finally, we examined whether the presence versus absence of maternal MDD history influenced report discrepancies for independent and dependent life events. Mothers with a history of MDD were more likely to over-report Dependent (*t* = 2.55, *p* = .01), but not Independent (*t* = 1.73, *p* = .09), events in their children’s lives than nondepressed mothers. Additionally, mothers with no history of MDD were more likely to under-report Independent life events relative to their children (*t* = 2.40, *p* = .02), but not Dependent life events (*t* = .65, *p* = .52).

Hierarchical linear regressions revealed that mothers with a history of MDD exhibited a greater degree of over-reporting youth Dependent stressors, independent of all designated covariates (Table 3). Further, mothers with no history of MDD were more likely to under-report Independent stressors in their children’s lives with the covariates included in the model (β = .11, *t* = 3.09, *p* < .01, *R* ^2^ = .66, Δ*R* ^2^ = .01, *f* ^2^ = .03). Both analyses utilized the same covariates as all other models (when controlling for youth reported events, we controlled for youth reported dependent and independent life events, respectively).

## Discussion

The aim of the current study was to examine the discrepancy in reporting of adolescents’ stressful events between mothers with and without a history of major depression. Prior literature highlights the problematic nature of reporter discrepancy due to a history of depression (De Los Reyes and Kazdin 2005). The main findings of the current study supported the hypothesis that maternal reporting of the stressful events of their children differed based on whether or not the mother had a prior diagnosis of depression. Consistent with the depression-distortion hypothesis (Richters and Pellegrini 1989), mothers with a prior diagnosis of depression reported more negative life events compared to their children than did nondepressed mothers. Although mothers with no history of depression were significantly more likely to report fewer events than their children only for independent life events, there was a consistent trend of reporting less events than their children for all discrepancy outcomes. Mothers with and without a history of depression exhibited a similar degree of absolute discrepancy from their children, indicating that the direction of discrepancy (over versus under) is the central distinction. Taken together, this suggests that mothers’ reporting of stressful events that occur in their children’s lives is discrepant based on the presence versus absence of a history of maternal depression.

The tendency for mothers with a history of MDD to over-report youth total stress (relative to their children) significantly more than nondepressed mothers may be driven by their tendency to over-report stress in the Family and Social domains. Although mothers may be expected to be accurate reporters of familial stress given their central role in family affairs, and adolescents may be expected to be better reporters of social stress given that parents are not observers in all social contexts, our results indicate that mothers with a history of MDD report more stressors in both of these domains than their children. Academic stressors, though trending in a similar direction as the other domains, may have exhibited less mother-youth discrepancy than social and family stressors because there are typically more objective indicators of youth academic stress readily available to both mothers and their children. For example, ALEQ Academic events such as “Got a report card with a C-average or less” and “Suspended or expelled from school” are more likely to be identified by both parties as occurring (e.g., the school would notify parents if the child was suspended) than many Family and Social events (e.g., “Friends pressured you to do things you didn’t want to do”). However, consistency across all three domains suggests a generalized tendency for mothers with a depression history to report more events relative to their children than nondepressed mothers.

Further, the tendency for mothers with a history of MDD to report more stressors that children contribute to (i.e., Dependent events) may suggest that maternal negative cognitive styles (inherent in depressive self-report biases) extrapolate to misappraisal of events when reporting on their children. Depressed individuals are more likely to self-focus (Pyszczynski et al. 1989), ruminate on negative life events (Nolen-Hoeksema 1991), and interpret neutral evaluations as negatively-valenced (Gotlib 1983). A similar schema of attributing events to personal deficits and characteristics may help explain why mothers with a history of MDD tend to report more stressors than their children that are in some way dependent on the child’s behaviors and characteristics. Our study illuminates that report discrepancies for youth-dependent stressors differ based on the presence versus absence of a maternal history of depression, but future research that incorporates objective measures of youth stress is necessary to investigate the mechanisms that may explain the relationship. Although mothers with no history of depression were more likely to under-report independent events relative to their children than mothers with a history of MDD, this discrepancy was not significantly exhibited overall, by content-domain, or for dependent stressors. Thus, replication is necessary to more strongly suggest that nondepressed mothers have a tendency to report less youth stress relative to their children.

This is the first study to extend the body of literature examining reporting discrepancy between parents and their children to the reporting of adolescents’ life experiences. Research has highlighted discrepancy between parent–child reporting of children’s psychopathology and behaviors, yet no research to date has examined this difference in the stress literature. Stressful events are consistently predictive of episodes of depression (Hammen 2005) and other forms of psychopathology (Grant et al. 2004). Upon close examination of the youth stress literature, researchers employ inconsistent methodologies in the collection of stress data that they then use to draw their conclusions. Although some researchers use only parent or only child reported life stress information, much research makes an attempt to combine both reports. The current study highlights that these attempts to understand the true occurrences of negative life events in youths’ lives may be discrepant based on a history of maternal depression. The potential consequence of utilizing discrepant data is inconsistent findings, which may amplify the role of stress as a risk factor for depression if life stress information does not take into account parental influence on reporting.

De Los Reyes and Kazdin (2005) proposed a theoretical framework to guide researchers in decreasing biases based on information discrepancy. The ABC Model attempts to incorporate the perspective of the reporter and combine data from multiple informants. This model is informed by the actor-observer phenomenon, memory recall research, and general heuristics that may influence biased information that leads to discrepant reports. Whereas this model was proposed to explain differences in the interpretation of behavior related to psychopathology, similar processes are likely in play when reporting negative life events. Individuals’ own attributions influence their memory and recall of events and thus may bias their contextual interpretation of events. Although we cannot determine biases from this study, it is possible that mothers with a history of MDD are hyperaware of youth stressors and are more accurate reporters; or, alternatively, they may be interpreting more events as stressful than objectively occurred.

However, inter-reporter discrepancies are thought to be conceptually linked with reporter biases, and studies have demonstrated that such discrepancies are associated with self-report biases inherent to depression and related constructs (De Los Reyes and Kazdin 2005). Subsequently, future research can examine if maternal negative cognitive styles (inherently regarded as perceptual biases) mediate the relationship between maternal depression and stress discrepancy scores when an objective measure of youth stress is available. Individuals with depression have exhibited self-report biases due to mood-congruent processing (Elliott et al. 2002), ruminative attention biases (Lavender and Watkins 2004), and negatively-biased autobiographical memories (Lyubomirsky et al. 1998). Extrapolation of these self-report biases to inter-reporter biases is a worthy endeavor to better understand the mechanisms underlying mother–youth discrepancies. Future research may also benefit from the use of the ABC Model’s theoretical framework as a translational tool to more adequately and systematically ascertain information from multiple sources.

Interpretation of the present results should take into account the relative strengths and limitations of the current study. This novel investigation of discrepant reporting of life stress utilized a large sample of racially diverse early adolescents and their mothers. Current adolescent and maternal depressive symptoms and diagnoses were controlled for to reduce the influence that current mood state had on reporting biases (Elliott et al. 2002; Gotlib 1983). Further, the number of youth reported events was controlled for, as a greater number of youth reported events statistically increase the likelihood of discrepancies with their mothers. This rationale was supported by our results, as mothers were less likely to report a greater number of events relative to their children (i.e., over-report) if their children reported more events overall. Thus, we employed very conservative tests of our hypotheses for all analyses. The prospective design incorporated interview based diagnoses of depression, and only mothers who met for a *DSM*-*IV*-*TR* diagnosis of MDD were identified as having a history of depression. Lewinsohn et al. (1981) proposed a scar hypothesis, whereby an episode of depression psychologically scars an individual in a way that increases their vulnerability to experience future distress. This scar may be evident in the current sample and influence the reporting of events, a postulation strengthened by the minimal variance accounted for by current maternal depressive symptoms and diagnoses. Although we expected that current maternal mood would be predictive of reporting discrepancy, it is possible that assessing for a history of maternal depression better captures the general influence of depression on reporting (i.e., scar hypothesis), whereas current mood state was assessed at a single time point and exhibited less variation across participants.

Alternatively, this study was limited by the use of checklists of negative life events. Although children are accurate informants about their own thoughts and feelings (Boyle et al. 1996), self-report checklists completed by mothers and children may be biased by various factors; thus, interpretation of the data should take this into account. Sources of biases can include social desirability (i.e., the participant is aware of a negative event, but withholding acknowledgement of the event so as not to be negatively judged by the researchers), memory recall deficits, and apathetic reporting (common in adolescents). Employing investigator-based contextual stress interviews (e.g., Hammen 2005) may be one option for reducing potential individual biases in future studies. Nonetheless, the use of self-reported stressors through a reliable and valid questionnaire is reasonable at this stage of investigation, as some research suggests that information derived from self-report checklists of stressors is comparable to information and associations obtained from life event interviews (Lewinsohn et al. 2003; Wagner et al. 2006). The current study hypothesized that nondepressed mothers would be more likely to under-report youth stress relative to the adolescents’ reports, and although this trend was consistent across analyses, only relative under-reporting of independent events was significantly different between groups. Furthermore, study results were based on the comparison of maternal reported events solely to adolescent reported events; ideally, future research could also incorporate the assessment of other informed individuals including fathers, teachers, and siblings.

Studying informant discrepancies in reporting life stress is important for researchers, clinicians, and families. Although life stress interviews are viewed as the ‘gold-standard’ method for obtaining information on events occurring in someone’s life, our results suggest the information gleaned from this approach may have important discrepancies and inconsistencies. Although researchers attempt to account for differences in reporting by combining reports, this may inevitably be a biased pursuit based on individual factors. The current research highlights the importance of taking into account individual factors that may influence reporting so that steps can be made to reduce such discrepancies (De Los Reyes and Kazdin 2005). Clinically, it may be important to ascertain accurate and in-depth histories of the child and family in order to more fully understand factors that may influence the reporting and interpretation of events. This study highlights a history of depression as a factor that may influence a parent to over-report events, but also calls attention to the possibility that mothers without such history may under-report events relative to their children. This may help clinicians to incorporate information from multiple sources more accurately and better interpret this information to influence treatment approaches and goals. Furthermore, an understanding of such discrepancies can be informative in developing adolescent treatment programs, as emphasizing parental involvement may benefit from additional training with parents on understanding their own interpretational predispositions. Finally, differences in the report and interpretation of events as stressful may influence parent–child and family functioning. A parent’s history of psychopathology may affect parenting practices as well as the perception of events as stressful. A more complete understanding of how parental characteristics influence their own views on the world may enhance their relationship with their children.
